# Serum albumin and activities of daily living in Chinese centenarians: a cross-sectional study

**DOI:** 10.1186/s12877-020-01631-7

**Published:** 2020-06-30

**Authors:** Ke Han, Shengshu Wang, Wangping Jia, Wenzhe Cao, Miao Liu, Shanshan Yang, Jianhua Wang, Yao He

**Affiliations:** 1 Department of Epidemiology, Institute of Geriatrics, the 2nd Medical Center, Chinese PLA General Hospital, 28 Fuxing Road, Beijing, 100853 China; 2 State Key Laboratory of Kidney Disease, Beijing Key Laboratory of Aging and Geriatrics, National Clinical Research Center for Geriatrics Diseases, the 2nd Medical Center, Chinese PLA General Hospital, 28 Fuxing Road, Beijing, 100853 China

**Keywords:** Activities of daily living, Serum albumin, Centenarians, Prevalence, China

## Abstract

**Background:**

Objective deterioration in activities of daily living (ADLs) exists among older population, and particularly worsens with age. Considering the criterion standard of positive aging and longevity, little information focusing on centenarians is available. This study set out to explore the relationship between serum albumin and ADLs among centenarians in long-lived areas.

**Methods:**

This population-based cross-sectional study investigated a full sample of Chinese centenarians in Hainan, the longest-lived area of China (*n* = 1002). We assessed serum albumin levels and basic and instrumental activities of daily living (BADLs and IADLs).

**Results:**

Of 1002 participants included in the analysis, 287 (28.64%) had BADL disabilities and 648 (64.67%) had IADL disabilities. The median level of serum albumin was 38.5 g/L (interquartile range, 36.2–41.3). The multivariable analyses controlling for socio-demographic characteristics, lifestyle, morbidities, and other influential factors showed that albumin level was associated with the total score of BADL (standard β = 0.335, *P* < 0.001) and IADL (standard β = 0.206, *P* < 0.001). With the increasing of albumin level, the risk of ADLs disability decreased (BADL: odds ratios [OR] = 0.835, 95% Confidence interval [CI]: 0.797–0.876; IADL: OR = 0.863, 95%CI: 0.824–0.905). In the stratified analyses, similar results were found in both sex, but were more prominent in women.

**Conclusions:**

Higher levels of serum albumin was a protective factor for the decline of ADLs in centenarians. This association can be observed in both genders and is more pronounced in women.

## Background

Activities of daily living (ADLs) are a core set of essential activities for individuals to live independently and is an important index that determines life expectancy and quality of life [1]. Physical disability is related to numerous adverse health outcomes such as cognitive impairment [2], mental disorder [3], decline in health-related quality of life [4], and increased burden of caregivers [5, 6]. The population of adults aged 85 years and older is growing faster than other groups; particularly, the number of centenarians has increased exponentially worldwide [7]. Objective deterioration of functional status exists among centenarians, and most of whom depend on help from family or professional aides in their ADLs [6]. The increase of centenarians may indicate a higher proportion of disabilities and poor health status in the older population, and eventually increase the burden of medical care and treatment [8].

As an important indicator of protein-energy malnutrition (PEM), hypoalbuminemia is very common among elderly people worldwide. Age increment leads to lower serum albumin (Alb) levels and a higher risk of hypoalbuminemia [9]. It has been demonstrated that hypoalbuminemia is associated with sarcopenia among the elderly [10]. Sarcopenia increases the risk of falls and fractures, which may eventually contribute to ADL disability. In addition, Alb-related chronic or acute inflammatory conditions may be correlated with the decline of ADLs in the elderly.

Studies have revealed that a strong relationship exists between Alb level and ADL in the general elderly population [9–19]. Given the potentially unique pattern of vulnerability, centenarians deserve more attention. However, few large-scale studies have been performed to examine its relationship among the oldest old individuals, and conclusions on gender differences remain controversial. In consideration of the high prevalence of hypoalbuminemia and functional decline with age, the association between Alb levels and ADLs deserves a more concentrated examination among centenarians.

Therefore, we assessed the distribution characteristics of Alb levels and ADL disability and investigated the association between Alb and ADL impairment especially the sex difference among centenarians using cross-sectional data obtained from a full sample of a centenarian cohort from Chinese longest-lived areas.

## Methods

### Study population

We used the baseline data of the China Hainan Centenarian Cohort Study (CHCCS) [20], a longitudinal observational study based on a full sample of the Hainan community-dwelling and institutionalized centenarians. Ethics approval was obtained from the Chinese People’s Liberation Army General Hospital Ethics Committee. All participants signed written informed consent.

According to the household registration provided by the Hainan Civil Affairs Bureau, there were 1811 living centenarians in total in 2014. After a rigorous age validation process, we established contact with 1473 eligible individuals. We also excluded individuals who were not conscious and could not perform ADLs or participate in the questionnaire survey, physical examination, and laboratory tests. Finally, 1002 centenarians aged 100–116 were enrolled into the study, and baseline data was collected from July 2014 to December 2016.

### Activities of daily living

We conducted face-to-face interviews to assess the ability to perform ADL and inspections through caregivers to ensure effective and reliable investigations. The Barthel index (BI) [21] and Lawton scale [22] were used to evaluate basic ADLs (BADLs) and instrumental ADLs (IADLs), respectively.

The BI was developed in 1965 and later revised to become a common instrument for measuring performance in activities of daily living [23, 24]. It includes 10 personal activities: feeding, grooming, bathing, dressing, toilet use, transfer between bed and wheelchair, mobility, climbing stairs, bowel care and bladder care [21]. Each item is scored 0–5 points, 0–10 points, or 0–15 points according to different evaluation results and summed to a total of 0–100 points. The total BI score is used to classify the individuals’ level of dependence as: 0–20 to total dependency, 21–60 to severe dependency, 61–90 to moderate dependency, and 91–99 to slight dependency. A total score of 100 indicates that the patient is independent of assistance from others [25]. In this study, we defined BADL disability as BI ≤60 points [26].

The Lawton scale is an appropriate tool for assessing independent living skills [27], which are considered more complex than BADL (using the telephone, food preparation, shopping, laundry, housekeeping, mode of transportation, handling finances, and handling own medications) [22]. Each skill measured by the scale requires some degree of both cognitive and physical function [28]. It takes 10–15 min to complete with a summary score ranging from 0 to 8. The total score of the scale sorts IADL dependence into 4 levels: severe (≤2), moderate (3–5), slight (6–7), and none (8). In this study, we defined IADL disability as a Lawton IADL score ≤ 2.

### Serum albumin

Experienced nurses collected 8 ml of blood from each participant in a fasting state using four vacutainer tubes (2 ml each). All blood samples were transported to the clinical laboratory in a refrigerated transport container (4 °C) and assayed in 4 h. Comprehensive metabolic panel examinations were performed using Cobas 8000 automatic biochemical autoanalyzer (Roche Products Ltd., Basel, Switzerland). Hypoalbuminemia is defined by a serum albumin < 35 g/L [29].

### Covariates

A trained interdisciplinary medical team carried out the questionnaire surveys and examinations. Face-to-face interviews were conducted in the participants’ residences or nearby clinics through appropriate regional dialects. The information was recorded in standardized structured questionnaires and entered into an electronic database with a cross-check by two persons. Socio-demographic characteristics assessed included age, gender, education, ethnicity, marriage, education, and type of residence. We also considered lifestyle characteristics, including smoking status, alcohol drinking status, and weekly exercise. Standing height and weight were measured using a manufactured instrument, and the body mass index (BMI) was calculated as weight/height^2^ (in kg/m^2^). Participants with a BMI < 18.5 kg/m^2^ were categorized as underweight [30]. The interviewees’ self-reported history of diagnosis and treatment of any specific medical condition was collected. Hypertension patients are defined as self-reporters or those with systolic blood pressure > 140 mmHg or diastolic blood pressure > 90 mmHg [31]. Similarly, diabetes patients are defined as self-reporters or those with fasting blood glucose concentrations ≥7.0 mmol/L [32]. Patients with impaired renal function are defined as self-reporters or those with glomerular filtration rate < 60 ml·min^− 1^/1.73 m^2^ [33]. In addition, the presence of heart disease and stroke was based solely on self-reports.

### Statistical analysis

Normally distributed continuous variables are described as mean ± standard deviation, and the non-normal distributions are described as median and interquartile range [IQR]. Categorical variables are expressed as n (%). Inter-group comparison were evaluated using the Mann–Whitney *U* test or the chi-squared test, as appropriate. Multivariable linear regression and logistic regression models were developed to evaluate the associations between Alb and the total score of ADLs. ADL disability (BI ≤60) and IADL disability (Lawton IADL score ≤ 2) were also used as outcomes and stratified analyses were performed. In the multivariable analyses, we considered socio-demographic characteristics, lifestyles, morbidity, and other influential factors including BMI classification, hemoglobin (Hb), total cholesterol (TC) level, and serum 25-hydroxyvitamin D (25OHD) as covariates. All statistical analyses were performed using SPSS Statistics version 24.0 (IBM Corporation, Armonk, NY, USA). All tests were two-sided and performed at a 5% significance level.

## Results

### Baseline characteristics

A total of 1002 centenarians were included in this study, including 822 women, who accounted for 82.04% of the total population. The ages of the participants ranged from 100 to 116 years, with a median age of 102 years (IQR, 101–104). The participants were mainly of Han ethnicity, illiterate, widowed/divorced/unmarried, and living with their families. The proportions of participants who smoked, drank alcohol, and exercised weekly were all lower than 20%. The median Alb level was 38.5 g/L (IQR, 36.2–41.3) for the entire sample. Participants with hypoalbuminemia had lower levels of Hb, TC, and 25OHD, and a lower prevalence of hypertension (Table 1).

**Table 1:** Prevalence of risk factors for ADL disability at two levels of serum albumin among 1002 centenarians

		Serum albumin		
Characteristics	Total(*n* = 1002)	Hypoalbuminemia (*n* = 174)	Normal Alb level (*n* = 828)	*P* value
Age, median (IQR),y	102.0 (101.0–104.0)	103.0 (101.0–105.0)	102.0 (101.0–104.0)	0.003
Female,%	822 (82.0)	140 (80.5)	682 (82.4)	0.551
Han ethnicity,%	883 (88.1)	156 (89.7)	727 (87.8)	0.492
Illiterate,%	915 (91.3)	162 (93.1)	753 (91.1)	0.325
Married,%	100 (10.0)	16 (9.2)	84 (10.1)	0.704
Living with families,%	863 (86.1)	159 (91.4)	704 (85.0)	0.027
Current smoker,%	35 (3.5)	6 (3.5)	29 (3.5)	0.963
Current alcohol drinker,%	99 (9.9)	14 (8.1)	85 (10.3)	0.002
Weekly exerciser,%	129 (12.9)	11 (6.3)	118 (14.3)	0.005
BMI < 18.5 kg/m^2^,%	575 (57.4)	118 (67.8)	457 (55.2)	0.005
Alb, median (IQR),g/L	38.5 (36.2–41.3)	32.8 (30.6–34.2)	39.4 (37.7–41.7)	<0.001
Hb, median (IQR), g/L	103.0 (114.0–123.0)	102.0 (94.0–113.0)	116.9 (107.0–125.0)	<0.001
TC, median (IQR), mmol/L	4.60 (4.1–5.3)	4.0 (3.4–4.6)	4.66 (4.2–5.3)	<0.001
25OHD, median (IQR), ng/mL	21.6 (16.9–27.8)	20.9 (14.0–26.5)	21.6 (17.4–28.1)	0.001
Diabetes,%	96 (9.6)	18 (10.3)	78 (9.4)	0.706
Hypertension,%	757 (75.6)	121 (69.5)	636 (76.8)	0.042
Heart disease,%	41 (4.1)	5 (2.9)	36 (4.3)	0.255
Stroke,%	22 (2.2)	1 (0.6)	21 (2.5)	0.153
Renal function impaired,%	299 (29.8)	56 (32.2)	243 (29.4)	0.457
BADL disability,%	287 (28.6)	94 (54.0)	193 (23.3)	<0.001
IADL disability,%	648 (64.7)	151 (86.8)	497 (60.0)	<0.001

Abbreviations: *ADL* Activities of daily living; *IQR* Interquartile range; *BMI* Body mass index; *Alb* Serum albumin; *Hb* Hemoglobin; *TC* Total cholesterol; *25OHD* Serum 25-hydroxyvitamin D

### Prevalence of ADL disability

The higher Alb group had a significantly lower rate of BADL and IADL disability than did the lower Alb group (Table 1). The prevalence of BADL disability was 28.64%, and that of IADL disability was 64.67%. Only 19 individuals were completely independent regarding IADL, and accounted for 1.9% of the population.

### Results of linear regression models

The standardized total score of BADL and IADL were included in the linear regression models as continuous variable respectively, and socio-demographic characteristics, lifestyle characteristics, morbidities, and other covariates were involved as adjust variables step by step. After adjustment, Alb was found to be significantly associated with ADLs (BADL: standard β = 0.335, *P* < 0.001; IADL: standard β = 0.206, *P* < 0.001, Table 2). The effect was weakened but in the same direction when the covariates were gradually incorporated. Similar results were found in both women (BADL: standard β = 0.347, *P* < 0.001; IADL: standard β = 0.205, *P* < 0.001, Table 2) and men (BADL: standard β = 0.218, *P* = 0.011; IADL: standard β = 0.194, *P* = 0.013, Table 2) but were more prominent in women.

**Table 2:** Association between serum albumin level and ADLs ^a, b^

	BADL			IADL		
	Standard β	Standard error	P	Standard β	Standard error	P
**Male**						
Crude model	0.241	0.017	0.001	0.292	0.021	<0.001
Model 1	0.236	0.018	0.002	0.273	0.020	<0.001
Model 2	0.230	0.020	0.006	0.200	0.022	0.009
Model 3	0.218	0.020	0.011	0.194	0.022	0.013
**Female**						
Crude model	0.405	0.008	<0.001	0.272	0.008	<0.001
Model 1	0.399	0.008	<0.001	0.255	0.008	<0.001
Model 2	0.360	0.009	<0.001	0.216	0.009	<0.001
Model 3	0.347	0.009	<0.001	0.205	0.009	<0.001
**Total**						
Crude model	0.373	0.007	<0.001	0.271	0.008	<0.001
Model 1	0.369	0.007	<0.001	0.256	0.007	<0.001
Model 2	0.344	0.008	<0.001	0.213	0.008	<0.001
Model 3	0.335	0.008	<0.001	0.206	0.008	<0.001

^a^ Crude model: None adjusted;

Model 1:Adjusted for age, ethnicity, marriage, educational levels, residence type

Model 2:Model 1 plus smoking status, alcohol drinking status, weekly exercise, BMI classification, hemoglobin, total cholesterol, serum 25-hydroxyvitamin D

Model 3:Model 2 plus diabetes, hypertension, heart disease, stroke, renal function impaired

^b^ ADLs were transformed to Z score with a mean of 0 and standard deviation (SD) of 1

### Results of logistic regression models

After adjustment, a significant association existed between Alb level and ADLs disability. The odds ratios (OR) of BADL disability for centenarians with one more g/L Alb was 0.835 (95% Confidence interval [CI]:0.797–0.876), and that of IADL was 0.863 (95%CI: 0.824–0.905). In stratified analyses by sex, the association in women (BADL: OR = 0.831, 95%CI: 0.788–0.877; IADL: OR = 0.863, 95%CI: 0.819–0.910, Table 3) was stronger than that in men (BADL: OR = 0.866, 95%CI: 0.768–0.977; IADL: OR = 0.888, 95%CI: 0.795–0.991, Table 3).

**Table 3:** Association between serum albumin level and BADL/IADL disability ^a, b^

	Total	Male	Female
BADL disability			
Crude model	0.829 (0.798,0.862)	0.899 (0.824,0.982)	0.813 (0.778,0.850)
Model 1	0.829 (0.797,0.862)	0.889 (0.809,0.977)	0.814 (0.779,0.851)
Model 2	0.832 (0.794,0.872)	0.864 (0.767,0.973)	0.827 (0.785,0.871)
Model 3	0.835 (0.797,0.876)	0.866 (0.768,0.977)	0.831 (0.788,0.877)
IADL disability			
Crude model	0.858 (0.826,0.892)	0.857 (0.788,0.932)	0.854 (0.818,0.892)
Model 1	0.856 (0.823,0.891)	0.861 (0.789,0.939)	0.855 (0.818,0.894)
Model 2	0.861 (0.822,0.903)	0.883 (0.793,0.983)	0.859 (0.815,0.905)
Model 3	0.863 (0.824,0.905)	0.888 (0.795,0.991)	0.863 (0.819,0.910)

^a^ The adjusted covariates in models are the same as in Table 2

^b^ Described as OR (95%CI)

## Discussion

Prior to our study, only one study had assessed the association between Alb level and ADL among Chinese centenarians [16]. Our data were derived from the 2014–2016 cross-sectional data of the CHCCS, which included a full sample of a large population in Hainan province. Hainan province has the highest proportion of centenarians (18.75/100000) and one of the highest average life expectancies (76.3 years in 2010) in China [34]. Therefore, our study may have some implications for positive aging and longevity.

As a clinical routinely-tested indicator and modifiable risk-related factor, Alb is an important and imperative potential target for public intervention and long-term clinical care [15]. Among 1002 Hainan centenarians assessed in this study, the average Alb level was 38.5 g/L (IQR, 36.2–41.3). This is lower than that identified in another Chinese longevity cohort with an average age of 97 (IQR, 96–98) years, which showed a mean albumin level of 42.4 ± 4.6 g/L [35]. The difference may be related to the age distribution characteristics of the subjects and differences in laboratory testing. A previous study showed that albumin concentration is related to aging and gradually decreases by 0.08–0.17 g/L per year, with levels tending to decline faster among males than among females [18]. In a study that included 95 Japanese centenarians, the mean Alb concentration was 36.0 ± 4.0 g/L among women and 36.0 ± 5.0 g/L among men [16]. The Alb concentration among centenarians was close to the lower limit of the adult reference value (35.0–50.0 g/L). Even in the high Alb group (with levels > 35.0 g/L), a significant proportion of the elderly was ADL disabled. Thus, we have reason to doubt the utility of using this traditional reference in the very old population, which is in line with the findings of Kuzuya et al. [36]. There is a need to establish a reference standard for healthy elderly populations, not only of Alb but also other significant serological indicators.

In this study, 287 (28.64%) centenarians were identified as having BADL disability. The prevalence of BADL disability was lower than that reported among older people and centenarians in similar studies regarding the population aged ≥90 years in other countries [37, 38]. Approximately 50% of participants were classified as having BADL disability in the baseline data of a local longevity cohort study, which investigated 433 long-lived individuals aged ≥95 years [35]. In the Chinese Longitudinal Healthy Longevity Survey, a nationwide survey covering about 85% of the total population of China, 37.1% of the sample had BADL disability at the baseline [39, 40]. One possible reason for the discrepancy is that diseases or factors of early death are delayed or absent among centenarians. Comparatively, long-lived individuals tend to live in good functional states. Besides, different versions of rating scales with different scoring criteria were used, and different definitions of disability were used among the studies.

BADL refers to an individual’s ability of independent living, while IADL focuses on more advanced skills in a specific social life. In our study, only 19 (1.9%) individuals had complete IADL independence according to the total score of the Lawton IADL scale. This means that almost every centenarian had lost at least one aspect of advanced functional independence necessary for daily living. The combination of all IADL items explains a larger portion of the difference in physical and psychological dimensions of quality of life compared to IADL items [41]. The performance of IADL in this sample is worse than that of the younger older population reported in similar studies [41–43]. This may be due to the fact that the incidence rate of ADL disability increases with age [44]. Advanced functions in daily life are important aspects of mental health and quality of life among older people. Thus, there is a need to pay attention to the impact of such a poor IADL in the very old.

The main finding of our study was that low levels of Alb were associated with ADL disability. Similar results have been reported in previous cross-sectional studies [11, 12, 15] and cohort studies [13, 17]. However, a few studies have reported negative results [45]. Alb plays an essential physiological role in the maintenance of health, including maintaining plasma colloid osmotic pressure and transporting endogenous substances and exogenous drugs. Its correlation with ADL may be attributable to the following hypotheses. As an essential indicator of nutritional status, Alb has great prognostic significance in protein-energy malnutrition. Also, it is a significant marker of acute and chronic inflammatory responses. Both hypoalbuminemia [10], inflammation [46] and malnutrition [47] are associated with reduced muscle mass (sarcopenia) in older adults. The positive association of sarcopenia with falls and fractures in older adults [48] is exact, which would contribute to the decline of ADL. Moreover, Alb plays a vital role in the pathogenesis of organ or system dysfunction/failure [49]. Albumin-associated inflammatory state and the associated poor acute pathological recovery might explain the ADL disability. On the other hand, IADL disability is a multidimensional construct that includes physical and cognitive domains. Significant cognitive decline is typical among older people [50] and is strongly associated with difficulties in IADL [51]. Thus, the severe deterioration of IADL among centenarians is explainable. The mechanism and causal link between Alb and ADL are not precise and need to be assessed in further studies.

Sex difference in this association were controversial in previous research. Our results are similar to the study of Okamura et al., which enrolled 797 men and 1047 women aged 60 to 74 [17]. However, a 3-year follow-up study showed that a combination of low Alb and low cholesterol levels was predictive of reduced functional performance, especially in men [45]. In a study of 97 male extremely longevous individuals (> 95 years old) conducted in China, researchers did not observe any association in men. Considering the very advanced level of age, our study recruited a relatively sufficient sample. Therefore, our research may provide some new evidence. However, we still could not determine if the results obtained in the male group are due to this fact or the influence of survivor bias. The crude mortality rate of elderly males is higher than that of females [52]. This may lead to the earlier death of the older men with severe impairment of ADL status and mask the relationship between Alb and impaired ADL. Therefore, more extensive research has to be conducted to understand the relationship entirely.

Limitations of the present study should be acknowledged. First, due to the cross-sectional design of the study, causal inference is limited. A longitudinal survey will further clarify the clinical significance and predictive effectiveness of Alb in the older population. Second, we excluded some individuals who were unable to participate in interviews and physical examinations before the investigation. The excluded subjects may be more likely in a low level of physical and cognitive function, which may lead to a certain underestimation of the prevalence of ADL disability. Third, the self-reported results of the questionnaire may be biased toward older people with poor cognitive function. Fourth, due to the lack of systematic nutritional assessment, we were unable to verify the exact association between Alb and nutritional status among centenarians.

## Conclusions

This study is the first to report on the levels of Alb and prevalence of ADL disability and to explore their association among Chinese centenarians. In this study, the concentration of Alb was low and the prevalences of BADL disability and IADL disability were 28.64 and 64.67%, respectively. Low levels of albumin were associated with a decline in ADL function among centenarians, and this association was gender-specific and more prominent among females. Paying attention to Alb levels among the elderly and properly caring and treating elderly persons with low Alb levels may significantly improve their physical function. However, there is a need to validate whether albumin-deficient interventions are ideal strategies for preventing disability development or improving the independence of older persons through appropriately designed randomized and controlled trials.

## Data Availability

Data can be obtained from the corresponding author upon reasonable request.

## Abbreviations

ADL: Activities of daily living
BADL: Basic activities of daily living
IADL: Instrumental activities of daily living
Alb: Serum albumin
CHCCS: China Hainan Centenarian Cohort Study
BMI: Body mass index
Hb: Hemoglobin
TC: Total cholesterol
25HOD: Serum 25-hydroxyvitamin D
IQR: Interquartile range
95% CI: 95% Confidence intervals
